# Rolling circle amplification (RCA) -based biosensor system for the fluorescent detection of miR-129-2-3p miRNA

**DOI:** 10.7717/peerj.14257

**Published:** 2022-10-24

**Authors:** Yan Ye, Yao Lin, Zilin Chi, Jiasheng Zhang, Fan Cai, Youzhi Zhu, Dianping Tang, Qingqiang Lin

**Affiliations:** 1 Fujian Normal University, College of Life Sciences, Fuzhou, Fujian, P. R. China; 2 Cooperation Base of Traditional Chinese Medicine-Oriented Chronic Disease Prevention and Treatment, Innovation and Transformation Center, Fujian University of Traditional Chinese Medicine, Fuzhou, China; 3 The First Affiliated Hospital of Fujian Medical University, Department of Thyroid and Breast Surgery, Fuzhou, Fujian, P. R. China; 4 Fuzhou University, Key Laboratory for Analytical Science of Food Safety and Biology (MOE & Fujian Province), State Key Laboratory of Photocatalysis on Energy and Environment, Department of Chemistry, Fuzhou, Fujian, P. R. China.

**Keywords:** RCA, Fluorescent detection, Biosensor, miR-129-2-3p

## Abstract

Herein, a versatile fluorescent bioanalysis platform for sensitive and specific screening of target miRNA (miR-129-2-3p) was innovatively designed by applying target-induced rolling circle amplification (RCA) for efficient signal amplification. Specifically, miR-129-2-3p was used as a ligation template to facilitate its ligation with padlock probes, followed by an RCA reaction in the presence of phi29 DNA polymerase. The dsDNA fragments and products were stained by SYBR Green I and then detected by fluorescence spectrophotometry. As a result, miR-129-2-3p concentrations as low as 50 nM could be detected. Furthermore, the expression of miR-129-2-3p in breast cancer patients was about twice that in healthy people. Therefore, the results indicated that the RCA-based biosensor system could be a valuable platform for miRNA detection in clinical diagnosis and biomedical study.

## Introduction

MicroRNAs (miRNAs) are a group of conserved, endogenous non-coding and short RNA (18–25 nucleotides) that regulate gene expression and play essential roles in cells, including proliferation, migration, differentiation, apoptosis and death (Xu et al., 2019; Gregory et al., 2004; Bartel, 2004; Sawyers, 2008; Ambros, 2001; Rossi, 2009). MicroRNAs negatively regulate gene expression via eliciting mRNA degradation or suppressing protein translation by targeting the 3′ or 5′ untranslated region (UTR) of the target gene (Luan et al., 2016; Shazadi et al., 2014, Bartel Wang et al., 2021). Past studies have found that miRNAs, as post-transcriptional regulators of gene expression, are closely associated with a variety of diseases, including cancer (Jiang et al., 2005; Luby & Zheng, 2017; Volinia et al., 2006), and are related to cancer initiation, progression and response to treatments (Brito et al., 2014; Schetter et al., 2008; Asaga et al., 2011). Accordingly, miRNAs extracted from serum or tumor tissue have been regarded as biomarkers for cancer diagnosis (Li et al., 2014; Cao et al., 2011; Krazinski et al., 2019).

MiR-129-2-3p is a member of the miR-129 family and is abnormally expressed in some tumors (Xiao et al., 2015; Kang et al., 2013; Lu et al., 2013; Tian et al., 2015; Yang et al., 2015; Tang et al., 2016), which is thought to have an inhibitory effect on various types of tumors (Gao et al., 2016).

MiR-129-2-3p plays a pivotal role in gastric cancer by restraining its migration and proliferation *in* *vitro* and slowing down gastric cancer growth *in* *vivo* via the inhibition of WWP1 (Ma et al., 2019; Yu et al., 2013a; Yu et al., 2013b). Moreover, some researchers also found that the expression of sex-determining region Y-box 4 (SOX4) was negatively correlated with the expression of miR-129-2-3p and miR-129-5p in gastric cancer (Yu et al., 2013a; Yu et al., 2013b). Overexpression of miR-129-2-3p significantly inhibits the proliferation and induces apoptosis of breast cancer cells (Tang et al., 2016). The expression levels of miR-129-2-3p in Ewing sarcoma tumor tissue samples are significantly lower than those in corresponding adjacent normal tissue samples (Tanoglu et al., 2021). The aberrant expression of the miR-129-2-3p is also detected in lung adenocarcinoma (Zhang et al., 2021). In human intrahepatic cholangiocarcinoma tissues and cell lines, the expression is notably decreased and the low expression of miR-129-2-3p is significantly correlated with distant metastasis and clinical stage (Huang et al., 2019).

Furthermore, some studies have also reported that miR-129-2-3p is associated with other human diseases. A previous study reported that miR-129-2-3p directly regulates the translation of two genes involved in inflammatory responses and apoptosis (Ccr2 and Casp6), and overexpression of miR-129-2-3p can promote wound healing in type 2 diabetic mice (Umehara et al., 2019). MiR-129-2-3p levels are significantly reduced in patients with ischemic stroke (IS) and are negatively associated with the risk of IS (Chen et al., 2020). The expression of miR-129-2-3p is up-regulated in cortical brain tissue and plasma of refractory temporal lobe epilepsy patients (Sun et al., 2016).

In the past few decades, some methods have been used to detect miRNA, including quantitative real-time polymerase chain reaction (qRT-PCR) (Chen et al., 2005), microarray (Thomson et al., 2004), northern blotting (Válóczi et al., 2004) and modified invader assay (Allawi et al., 2004). Some new detection methods have recently been invented, such as representative loop-mediated isothermal amplification (LAMP) (Li et al., 2011) and rolling circle amplification (RCA) (Xu et al., 2018). In this study, an RCA-based biosensor system was used to perform the amplification detection of miRNA (miR-129-2-3p) *in vitro*. This method achieves signal amplification and biosensing, which has exciting potential in clinical diagnosis.

## Materials & Methods

### Materials

The DEPC Treated Water (DEPC-H_2_O) and deoxyribonucleotides mixture (dNTPs) were purchased from Sangon Biotech (Shanghai, China). Phi29 DNA polymerase (10,000 U/mL) and 10 ×phi29 DNA polymerase reaction buffer, SYBR Green I were obtained from Thermo Fisher Scientific (Shanghai, China). T4 DNA ligase and 10 × T4 DNA ligase reaction buffer were provided by TaKaRa Biotechnology Co., Ltd. (Dalian, China). The padlock probe and oligonucleotides in this study were synthesized and PAGE purified by Sangon Biotech (Shanghai, China), the padlock probe was modified with the 5′-phosphate group. The sequences (5′–3′) were shown in Table 1.

**Table 1:** Sequences used in this study.

Serial number	Name	Sequence
1	miR-129-2-3p	AAGCCCUUACC CCAAAAAGCAU
2	3p-A	CAGCCCUUACC CCAAAAAGCAU
3	3p-B	AAGCCCUUUCC CCAAAAAGCAU
4	3p-C	AAGCCCUUACG GCAAAAAGCAU
5	3p-D	AAGCCCUUACC UCAAAAAGCAU
6	3p-E	AAGCCCUUACC CCAAAAAGCAA
7	Padlock probe 1(PP1)	GGTAAGGGCTTAAATCAACCGTACGGCTCAAACGCATGCTTTTTGG
8	Random padlock probe 1(RP1)	GGTAAGGGCTTAAACCTCAAGTCTACCAAGGACGCATGCTTTTTGG
9	Random padlock probe 2(RP2)	GGTAAGGGCTTAAATCAACGAGCGTCCTCAAACGCATGCTTTTTGGTATACAAC
10	Padlock probe 2 (PP2)	GGTAAGGGCTTCCGTACGGACAACCTACTACCTCACCGTACGGCTATA CCTACTACCTA CCGTACGGATGCTTTTTGG

### Ligation reaction of padlock probe and miRNA

The ligation was carried out in 10 µL of the reaction system. 1 µL of 10 × T4 DNA ligase buffer, 2 µL of 10 µM padlock probe, 2 µL of miRNA, and 4 µL of H_2_O were added to a PCR tube and heated at 90 °C for 3 min for annealing reaction, and then slowly cooled down to room temperature (RT). Subsequently, 350 U/mL T4 DNA ligase (1µL) was added to the reaction solution and incubated at RT for 3 h.

### The RCA reaction of miRNA

After the ligation reaction, 2 µL of 10 mM dNTP (1 mM), 2 µL of 10 × phi29 DNA buffer, 5.7 µL of H_2_O, and 0.3 µL of phi29 DNA polymerase (3U) were added to the tube and then kept at 30 °C for 3 h to induce the RCA reaction. Finally, the enzymatic reaction was stopped by maintaining the temperature at 65 °C for 10 min. RCA reaction was carried out in T1 Thermocycler (Biometra, Jena, Germany).

### Fluorescence detection

To detect the fluorescence of hybridization events of target/linear padlock probes, 6 µL RCA product and 2 µL of 100 × SYBR Green I were mixed, then incubated at RT for 30 min and diluted to a final volume of 200 µL with DEPC-H_2_O. The fluorescent spectra were detected by the Hitachi F-7000 fluorescence spectrometer (Hitachi, Ltd., Tokyo, Japan) at RT. The excitation wavelength was set to 480 nm with an emission range of 500 nm–700 nm. A fluorescence peak emission wavelength of 550 nm was recorded to evaluate the capability of our system.

### Gel electrophoresis analysis

The nucleic acids produced by the RCA reaction were analyzed by PAGE (polyacrylamide gel electrophoresis). Firstly, the dye was pre-mixed with 100 µL DiGelRed, 50 µL loading dye, and 2 µL cyber gold, then a 20 µL aliquot of RCA product solution was mixed with 20 µL mixed dye solution. Subsequently, 5 µL of the resulting solution load was placed into the lane for 30% PAGE. Gel electrophoresis was performed for 30 min at 195 V in a 5 × TBE solution. The ChemiDoc XRS imaging system (BIO-RAD, USA) was used to visualize the gel images.

### Extraction of microarray gene expression data from breast cancer patient datasets

The serum microarray datasets of breast cancer patients were extracted from Gene Expression Omnibus (GEO) database (http://www.ncbi.nlm.nih.gov/geo/). GSE73002 included 1670 breast cancer patients and 2,682 healthy volunteers. Receiver operating characteristics (ROC) curve analysis was performed to analyze the ability of miR-129-2-3p as a serum biomarker for breast cancer. ROC curve was generated with SPSS software.

### Patients and specimens

The research consisted of 6 breast cancer samples and six healthy volunteer samples. All the patients under went breast resection at the First Affiliated Hospital of Fujian Medical University between January 2020 and June 2021. The inclusion criteria for patients were: (1) histologically confirmed breast cancer; (2) no history of other malignancy; (3) no prior neoadjuvant chemotherapy. The study was performed with the approval of the Ethics Committee of the First Affiliated Hospital of Fujian Medical University. Written informed consent was obtained from the patients, and specimens were stored in the hospital database and used for research.

## Results

### Extraction of microarray gene expression data from breast cancer patient datasets

**Figure 1:**
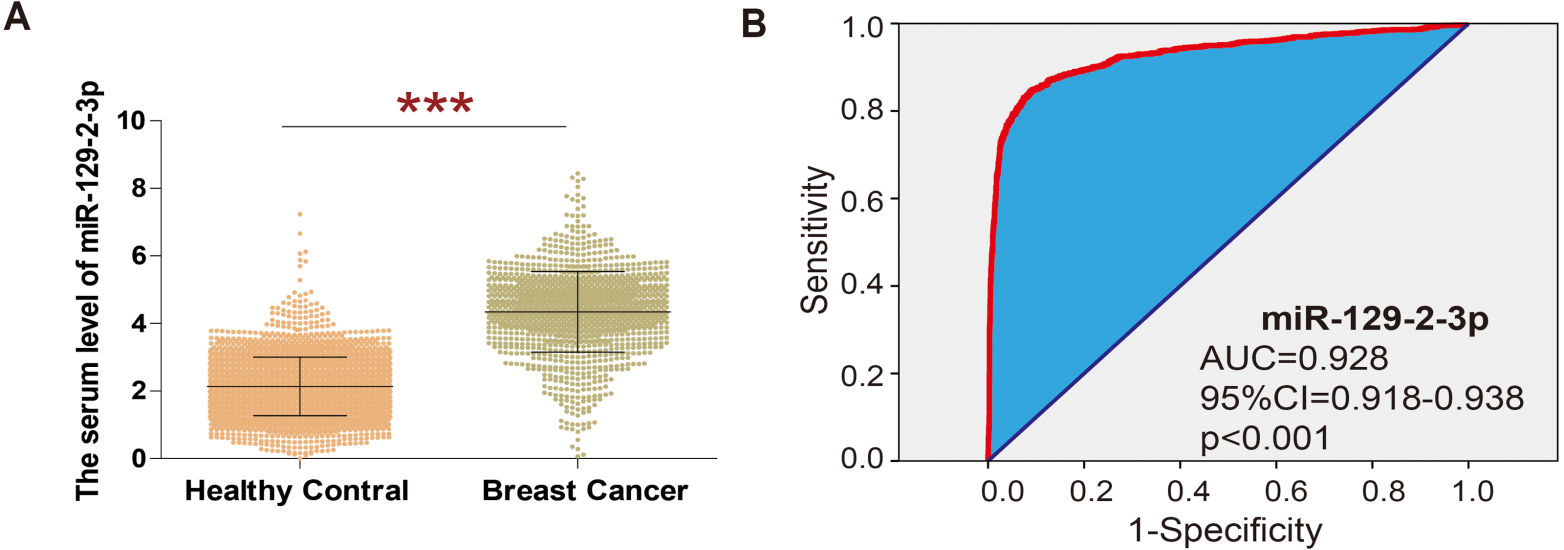
Extraction of microarray gene expression data from breast cancer patient datasets. (A) The analysis of GSE73002 data sets showed that in breast cancer, the level of miR-129-2-3p was significantly higher than the healthy volunteers. (B) ROC curve analysis showed that expression of miR-129-2-3p has potential diagnostic value for breast cancer.

In order to explore the expression of miR-129-2-3p in breast cancer, GSE73002 datasets were analyzed, revealing that the level of miR-129-2-3p was significantly higher in breast cancer than in healthy volunteers (shown in Fig. 1A). ROC curve analysis showed that miR-129-2-3p expression has potential diagnostic value for breast cancer. Data from the GSE73002 dataset showed that miR-129-2-3p may be an important diagnostic factor for breast cancer (Area Under Curve (AUC) = 0.928; 95% Cl [0.918–0.938]; *p* < 0.001; Fig. 1B).

### The feasibility of RCA-based biosensor system strategy

**Figure 2:**
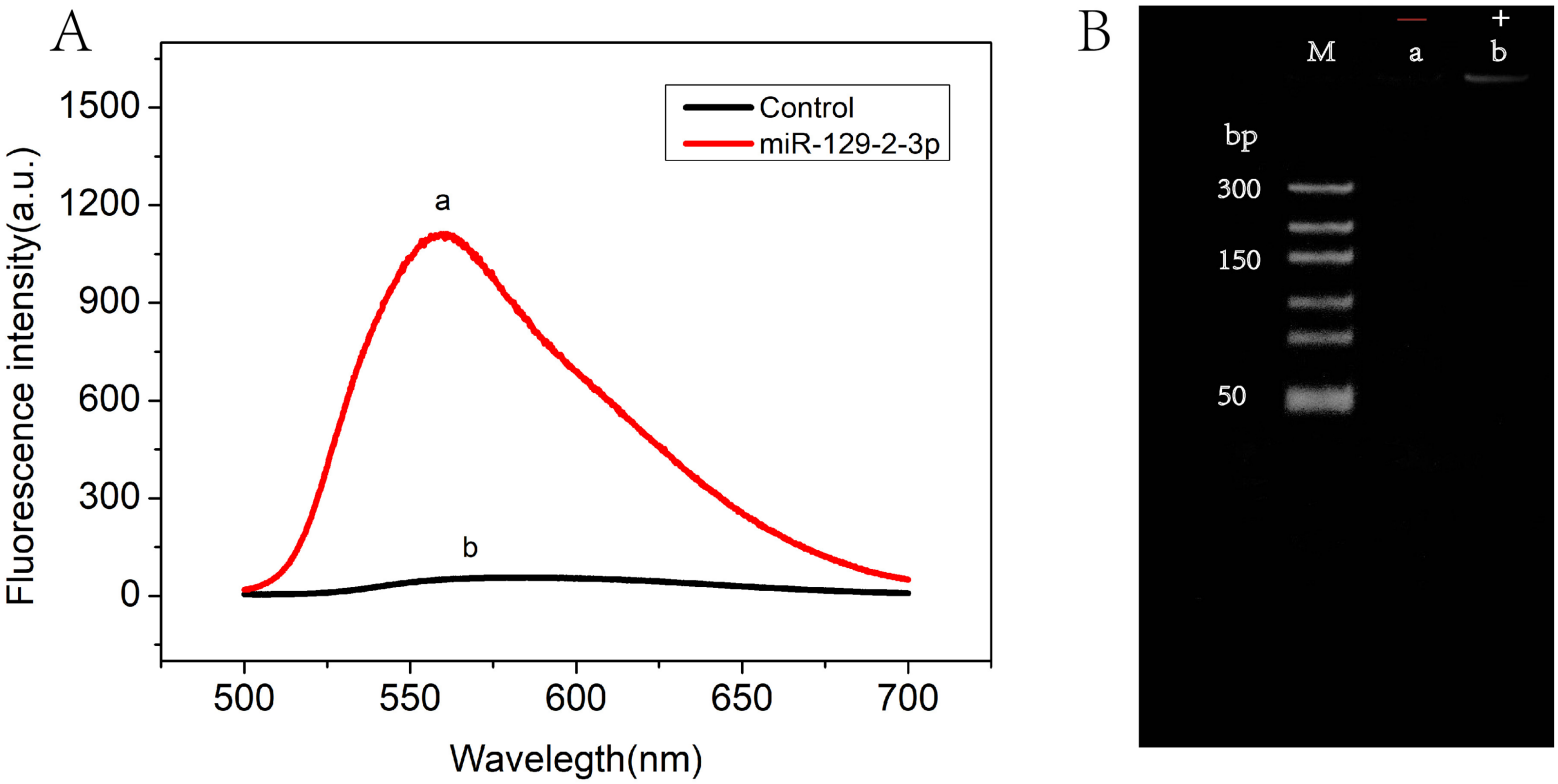
The feasibility of RCA-based biosensor system. (A) Fluorescence spectra of RCA-based biosensor system in the absence (b) and presence (a) of target miR-129-2-3p; (B) PAGE gel electrophoresis results for the amplification products of absence (a) and presence (b) of target miR-129-2-3p.

The feasibility of the RCA-based biosensor system was verified with fluorescence spectral characteristics. Figure 2A showed the fluorescence emission spectra in the absence (curve b) and presence (curve a) of miR-129-2-3p. Low fluorescence intensity was observed in solutions without miR-129-2-3p. On the contrary, the fluorescence intensity was significantly enhanced after miR-129-2-3p was introduced into the solutions (curve a). These results indicated that miR-129-2-3p could induce ligation reactions, followed by RCA reactions, subsequently leading to the production of a large number of dsDNA fragments. To further explore the feasibility of our strategy, PAGE gel electrophoresis was performed, as shown in Fig. 2B. No bands were observed in the absence of miR-129-2-3p (lane a), but a distinct band appeared in the presence of miR-129-2-3p (lane b). Therefore, the results of fluorescence spectral characteristics and PAGE gel electrophoresis suggested that the RCA-based biosensor system can be used to detect miR-129-2-3p.

### Optimization of experimental conditions

**Figure 3:**
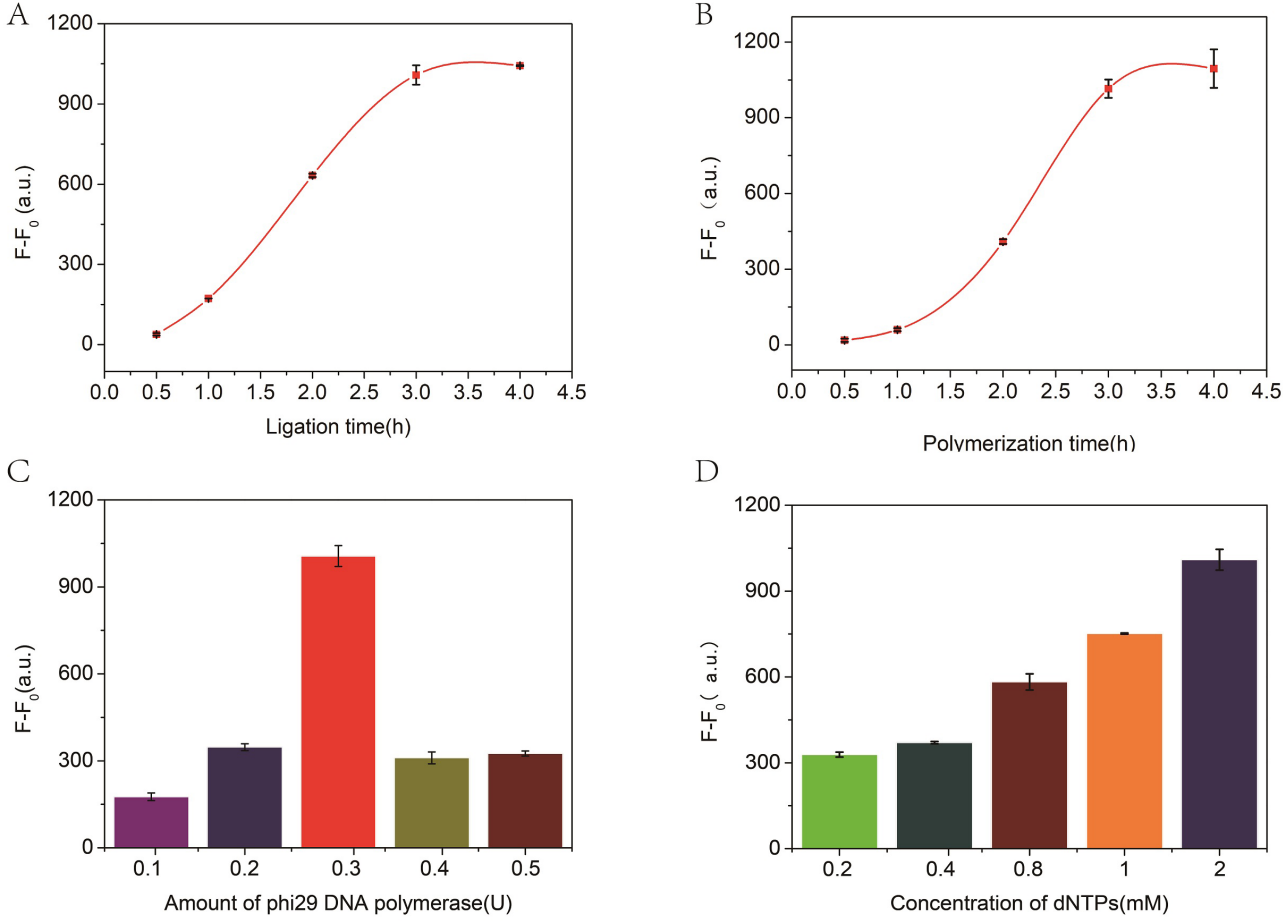
The relationship between fluorescence intensity and ligation time (A), polymerization time (B), and the concentration of phi29 DNA polymerase (C) and dNTPs (D). F and F_0_ were the fluorescence intensity induced by target miRNA and Blank, respectively. The error bar was calculated from two independent experiments.

The incubation time for ligation and amplification and the concentration of phi29 DNA polymerase and dNTPs play a significant role in these experiments. Therefore, the experimental conditions were optimized to achieve the best performance. As illustrated in Fig. 3A, with the increase of ligation time, the fluorescence changes gradually increased and to stabilized within 3 h. Therefore, a 3 h ligation time was chosen for further experiments. Similarly, the number of RCA reaction products is closely related to the polymerization time, and the measured values are shown in Fig. 3B. Thus, the RCA reactions lasts for 3 h in the proposed biosensor system. Subsequently, the effects of the amount of phi29 DNA polymerase and the concentration of dNTPs on signal intensity were evaluated to further evaluate the biosensor system. All measured data are shown in Figs. 3C and 3D, respectively. Consequently, 2 mM dNTP and 3 U of phi29 DNA polymerase were used in subsequent experiments.

### Sensitivity of RCA-based biosensor system for miRNA detection

**Figure 4:**
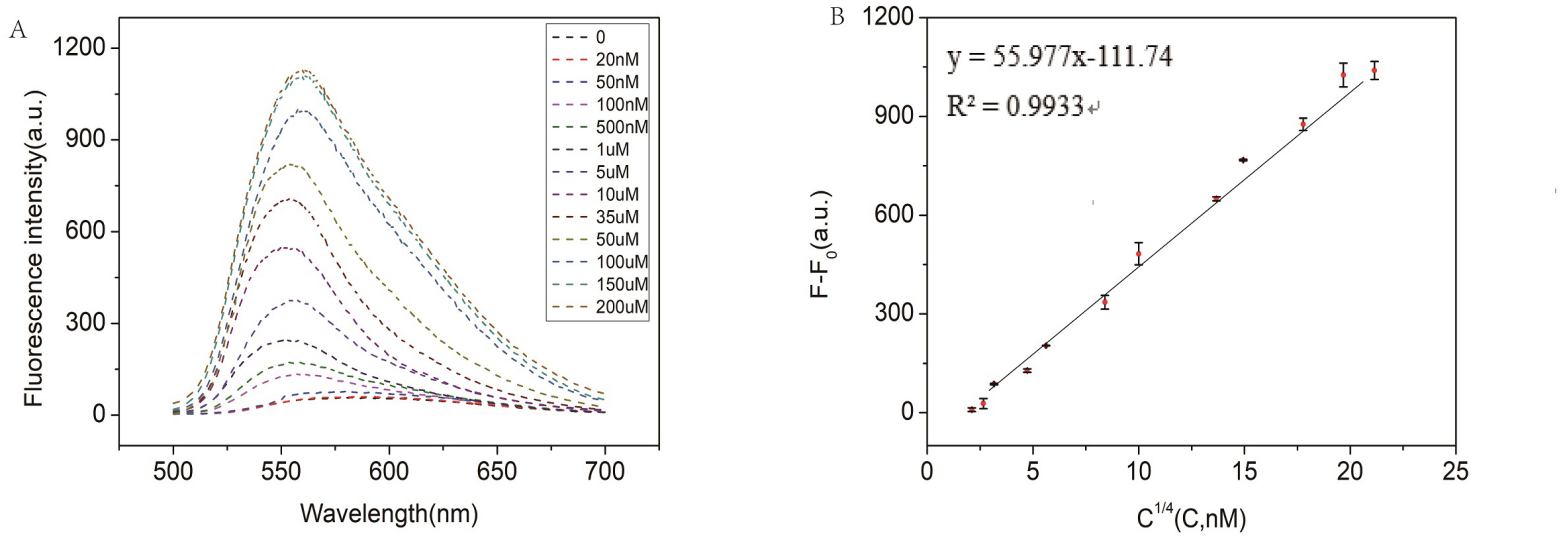
(A) Fluorescence intensity of miR-129-2-3p miRNA at different concentrations; (B) linear relationship between fluorescence intensity ratio (F-F_0_) and target miR-129-2-3p miRNA concentration, from 50 nM to 200 µM. F and F_0_ were the fluorescence intensity induced by target miRNA and Blank, respectively. The error bar was calculated from two independent experiments.

In order to test the capability of the RCA-based biosensor system for miRNA detection, different concentrations of miR-129-2-3p solution were detected. As shown in Fig. 4A, the fluorescence intensity increased with the increase of miR-129-2-3p concentration within 0–150 µM. It indicated that the change in fluorescence intensity reflected the concentration of miR-129-2-3p. As illustrated in Fig. 4B, there was a significant linear relationship between the fluorescence signal and target concentration in the range of 20 nM to 150 µM, and the correlation coefficient R^2^ was 0.9933. The detection limit (LOD) of the aptasensor was estimated to be 50 nM (S/N = 3). This is the first time that an RCA-based biosensor system has been used to detect miR-129-2-3p, which is expected to provide a sensitive detection method for miR-129-2-3p as a target marker in clinical practice.

**Figure 5:**
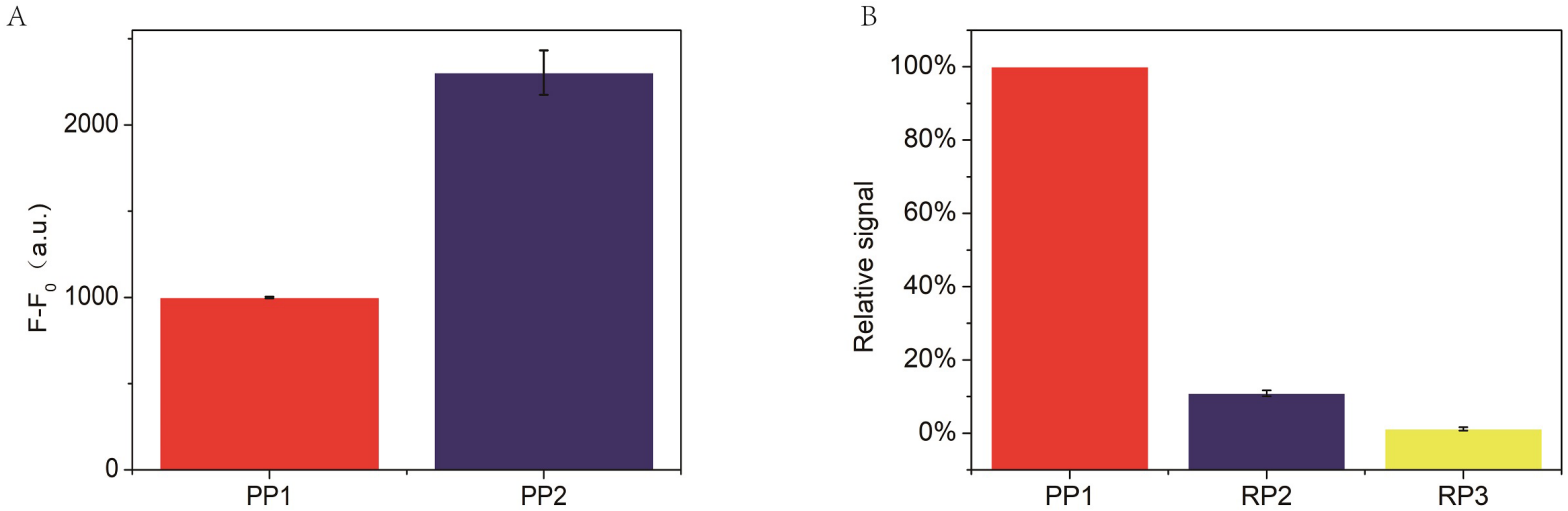
(A) Comparison of padlock probe 1(PP1) to miR-129-2-3p with padlock probe 2(PP2), where F and F_0_ are the fluorescence intensity corresponding to miR-129-2-3p and blank, respectively. (B) Comparison of padlock probe 1(PP1) to miR-129-2-3p with random padlock probe 1 and 2. The relative signal is estimated from (Fb-Fb_0_)/(Fa-Fa_0_) × 100. Fb and Fb_0_ are the fluorescence intensity in the presence and absence of random padlock probe1 and 2(PP1 and PP2), respectively, while Fa and Fa_0_ are the fluorescence intensity in the presence and absence of miR-129-2-3p, respectively. In this section, the relative signal of miR-129-2-3p is established as 100 (%). The error bar was calculated from two independent experiments.

In the RCA-based biosensor system, the fluorescence signal is caused by SYBR Green I interacting with the dsDNA fragments, which determines the fluorescence signal intensity. Therefore, the padlock probe 2 was designed containing three palindromes to indicate the relationship between the fluorescence signal and the palindromic fragment number. Moreover, two random padlock probes without palindromes were designed to indicate the relationship between the fluorescence signal and the palindromic fragment number. As shown in Fig. 5A, the fluorescence intensity increased about 2 times when the number of palindrome fragments increased to three. As illustrated in Fig. 5B, the fluorescence signal of the two random padlock probes was lower than that of padlock probe1. It seemed that the performance of the RCA-based biosensor system for miR-129-2-3p detection could be further improved by optimizing the number of palindrome fragments.

### Detection specificity of miR-129-2-3p

**Figure 6:**
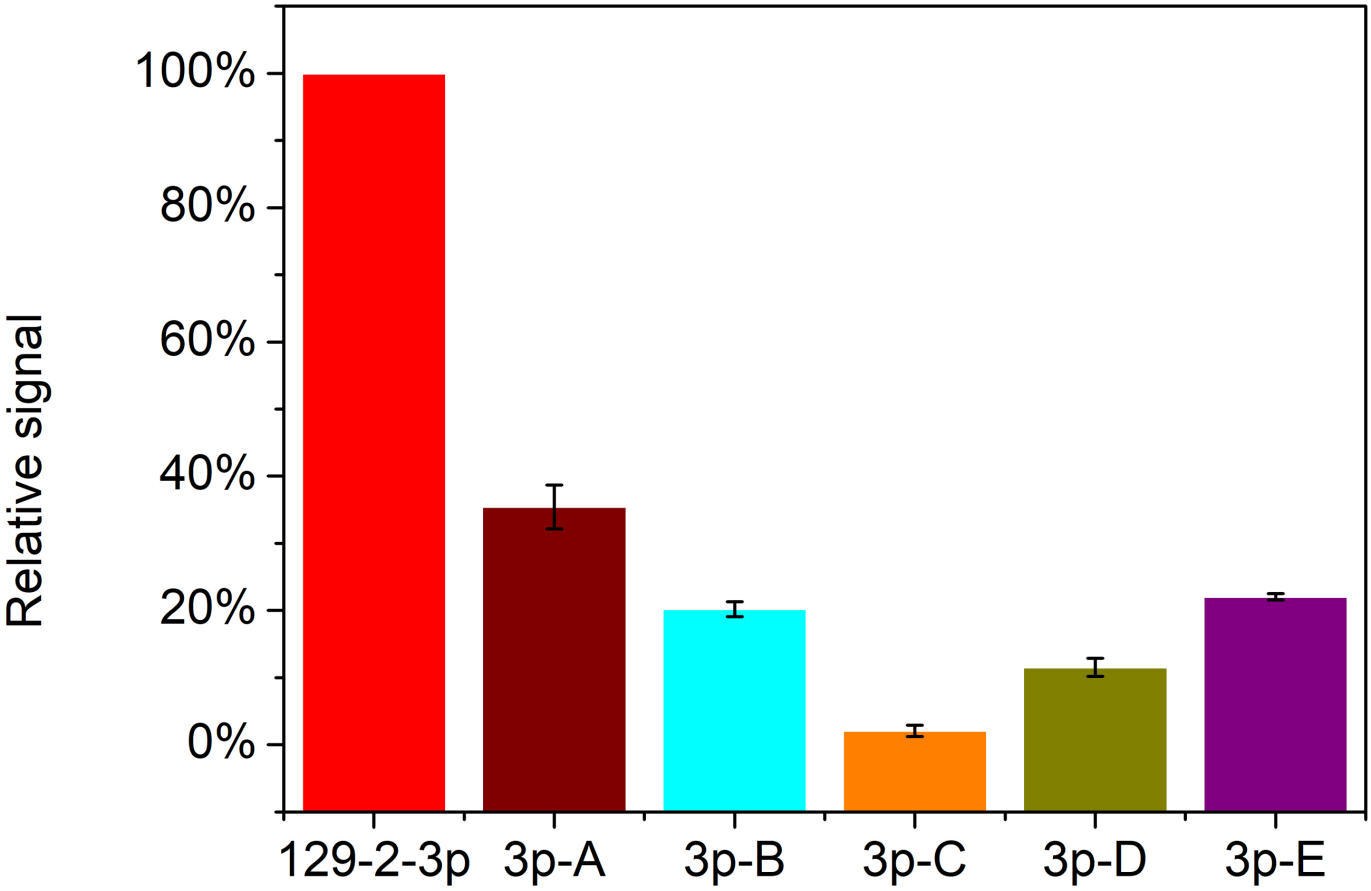
Detection specificity of the RCA-based biosensor system toward miR-129-2-3p over other mutated miRNAs. The relative signal is estimated from (Fb-Fb_0_)/(Fa-Fa_0_) × 100. Fb and Fb_0_ are the fluorescence intensity in the presence and absence of mutated miRNAs, respectively, while Fa and Fa_0_ are the fluorescence intensity in the presence and absence of miR-129-2-3p, respectively. In this section, relative signal of miR-129-2-3p is established as 100 (%). The error bar was calculated from two independent experiments.

Apart from the sensitivity of detection, specificity is another key factor for the application of this strategy for miR-129-2-3p analysis. The specificity of detection for miRNA is of great significance due to the short length and similar base sequence of miRNAs. So, five mutations of miR-129-2-3p (3p-A, 3p-B, 3p-C, 3p-D and 3p-E) and miR-129-2-3p were used to assess the detection specificity of the RCA-based biosensor system. As shown in Fig. 6, only target miR-129-2-3p elicited a high fluorescence signal. In contrast, the other five mutated miRNAs only induced slight signal changes: 33.1%, 19.4%, 1.5%, 10.6%, and 21.7%, respectively, compared with perfectly matched miR-129-2-3p. Therefore, the padlock probe1 in this work could specifically hybridize with miR-129-2-3p and promote subsequent reactions. The RCA-based biosensor system can be used to distinguish miR-129-2-3p from other non-target miRNAs.

### Detection of real samples

**Figure 7:**
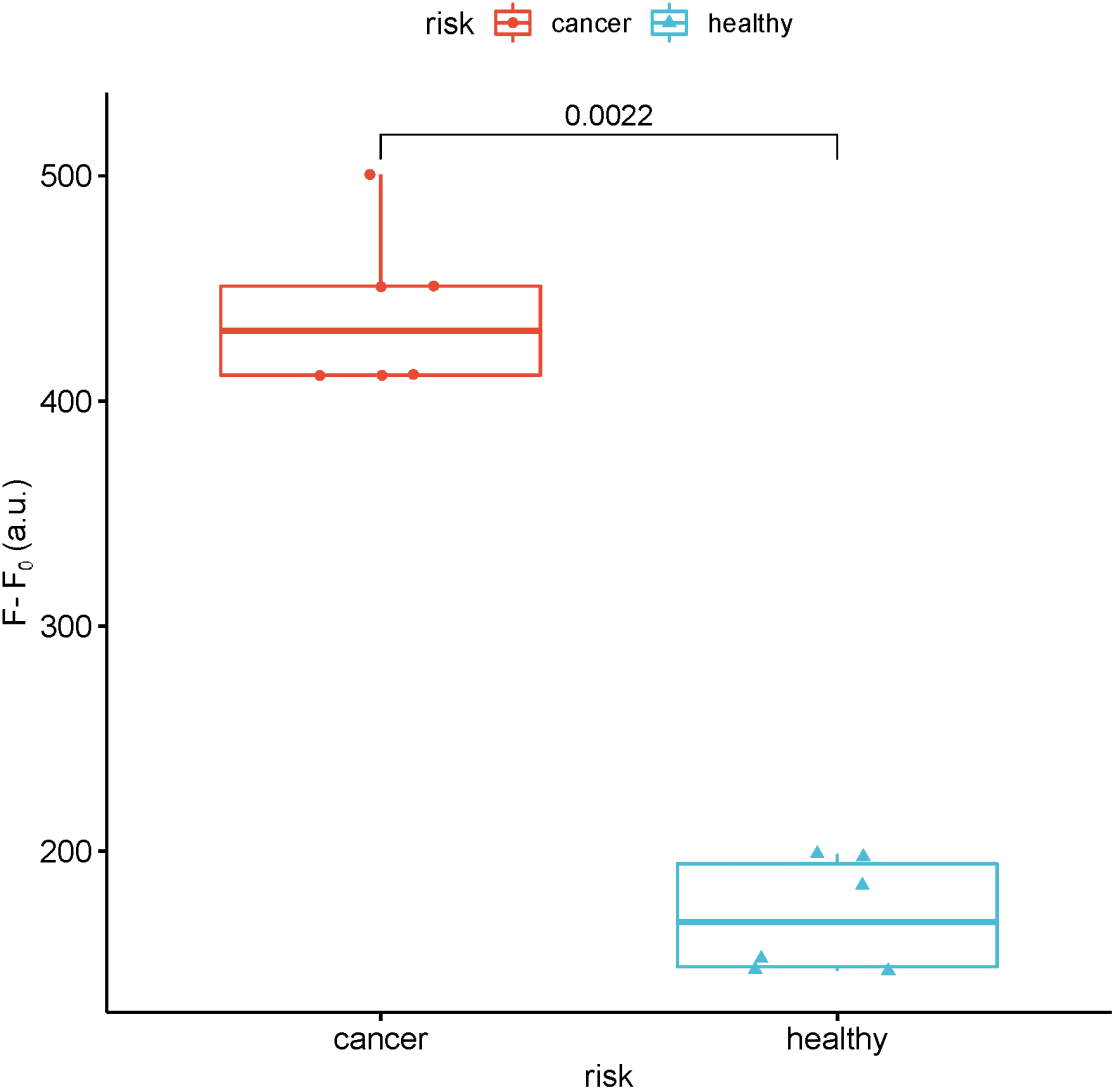
Detection of the fluorescence intensity of miR-129-2-3p in the serum of breast cancer patients and healthy people. F and F_0_ were the fluorescence intensity induced by sample and Blank, respectively.

To study the feasibility of this method in real sample analysis, the fluorescence intensity of miR-129-2-3p was detected in the serum of breast cancer patients and healthy people. As shown in Fig. 7, the fluorescence intensity of miR-129-2-3p in breast cancer patients is about twice that of healthy people.

## Discussion

MiR-129-2-3p is a member of the miR-129 family, and its abnormal expression is frequently detected in tumors; MiR129-2-3p is thought to have an inhibitory effect on various types of tumors. Over the past decades, different methods were used to detect miRNAs, including quantitative real-time polymerase chain reaction (qRT-PCR) (Chen et al., 2005), microarray (Thomson et al., 2004), northern blotting (Válóczi et al., 2004) and modified invader assay (Allawi et al., 2004). However, these methods still have some limitations in clinical diagnosis. For example, PCR may affect gene expression. Northern blotting is a time-consuming process with low sensitivity. Microarray cannot be used due to high cost, lower sensitivity, and poor reproducibility. Herein, a versatile fluorescent bioanalysis platform for sensitive and specific screening of target miRNA (miR-129-2-3p miRNA) was innovatively designed by using target-induced rolling circle amplification (RCA) for efficient signal amplification.

The principle of the RCA-based biosensor system for detecting target miRNA is described in Scheme S1. This system consists of padlock probe 1 (including a palindrome sequence) complementary to the sequence of the target miRNA, target miRNA, ligase and polymerase, and SYBR Green I. In the presence of target miRNA, cyclized padlock probe 1 is obtained with the help of T4 DNA ligase. The RCA polymerization reaction is initiated in the presence of phi29 DNA polymerase and dNTPs. As a result, the RCA reaction produced a long single stranded DNA, many copies of dsDNA fragments of the target because of the self-hybridization of the palindromic sequences. Subsequently, the dsDNA binds to SYBR Green I, and the fluorescence signal can be detected by the fluorescence spectrometer. Through this method, as long as target miRNA and padlock probe1 are connected during the reaction, a large number of dsDNA fragments can be produced after RCA. Since the RCA products are long dsDNA, SYBR Green I is an asymmetrical cyanine dye used as a nucleic acid stain to enhance the fluorescence intensity. Therefore, the RCA-based biosensor system is likely to provide good sensitivity for miR-129-2-3p detection. As a result, miR-129-2-3p miRNA concentrations as low as 50 nM can be detected. In the analysis of real samples, the fluorescence intensity of miR-129-2-3p in breast cancer patients is about twice that in healthy people. Therefore, the results indicated that the RCA -based biosensor system has the potential to become a valuable platform for miRNA detection in clinical diagnosis and biomedical study.

## Conclusions

In summary, we have developed a specific fluorescent detection method for miR-129-2-3p using a palindromic padlock probe in an RCA-based biosensor system. Target miRNA is used as a polymeric primer to hybridize with the padlock probe. The RCA reactions can easily occur in the presence of polymerases, which produces a large number of dsDNA fragments. SYBR Green I intercalates the dsDNA fragments, and the fluorescence signal is detected. Utilizing this RCA-based biosensor system, miR-129-2-3p can be detected at a concentration as low as 50 nM with a good linear response range, even using only one palindromic padlock probe. In the analysis of real samples, the expression of miR-129-2-3p in breast cancer patients was about twice that of healthy people. This research highlights the potential of this sensing system to detect miR-129-2-3p as a tumor biomarker in cancer diagnosis and prognosis. It also offers a new amplification technique for the biological studies of miR-129-2-3p.

##  Supplemental Information

10.7717/peerj.14257/supp-1 Supplemental Information 1 Raw data for Fig. 1

10.7717/peerj.14257/supp-2 Supplemental Information 2 Principle of RCA-based biosensor system for miR-129-2-3p detection Hybridization of target to padlock probe by annealing. Circular padlock probe is formed via ligation in the presences of T4 DNA ligase. RCA reaction occurs and target miRNA is amplified to produce a large amount of dsDNA. As a result, dsDNA are able to bind SYBR Green I, then fluorescence signal can be read.

10.7717/peerj.14257/supp-3 Supplemental Information 3 Raw data for Fig. 2 The feasibility of RCA-based biosensor system. (A) Fluorescence spectra of RCA-based biosensor system in the absence (b) and presence (a) of target miR-129-2-3p; (B) PAGE gel electrophoresis results for the amplification products of absence (a) and presence (b) of target miR-129-2-3p.

10.7717/peerj.14257/supp-4 Supplemental Information 4 Raw data for Fig. 3 The relationship between fluorescence intensity and ligation time (A), polymerization time (B), and the concentration of phi29 DNA polymerase (C) and dNTPs (D). F and F0 were the fluorescence intensity induced by target miRNA and Blank, respectively. The error bar was calculated from two independent experiments.

10.7717/peerj.14257/supp-5 Supplemental Information 5 Raw data for Fig. 4 (A) Fluorescence intensity of miR-129-2-3p miRNA at different concentrations; (B) Linear relationship between fluorescence intensity ratio (F-F_0_) and target miR-129-2-3p miRNA concentration, from 50 nM to 200 *μ*M. F and F_0_ were the fluorescence intensity induced by target miRNA and Blank, respectively. The error bar was calculated from two independent experiments.

10.7717/peerj.14257/supp-6 Supplemental Information 6 Raw data for Fig. 5 (A) Comparison of padlock probe 1 to miR-129-2-3p miRNA with padlock probe 2, where F and F_0_ are the fluorescence intensity corresponding to miR-129-2-3p and blank, respectively. (B) Comparison of padlock probe 1 to miR-129-2-3p miRNA with random padlock probe1 and 2, The relative signal is estimated from (Fb-Fb_0_)/(Fa-Fa_0_) × 100. Fb and Fb _0_ are the fluorescence intensity in the presence and absence of random padlock probe1 and 2, respectively, while Fa and Fa_0_ are the fluorescence intensity in the presence and absence of miR-129-2-3p, respectively. In this section, relative signal of miR-129-2-3p miRNA is established as 100 (%). The error bar was calculated from two independent experiments.

10.7717/peerj.14257/supp-7 Supplemental Information 7 Raw data for Fig. 6 Detection specificity of the RCA-based biosensor system toward miR-129-2-3p over other mutated miRNAs. The relative signal is estimated from (Fb-Fb_0_)/(Fa-Fa_0_) × 100. Fb and Fb_0_ are the fluorescence intensity in the presence and absence of mutated miRNAs, respectively, while Fa and Fa _0_ are the fluorescence intensity in the presence and absence of miR-129-2-3p, respectively. In this section, relative signal of miR-129-2-3p miRNA is established as 100 (%). The error bar was calculated from two independent experiments.

10.7717/peerj.14257/supp-8 Supplemental Information 8 Raw data for Fig. 7

## Additional Information and Declarations

### Data Availability

The following information was supplied regarding data availability: The raw data is available in the Supplementary Files.
