# Supplementary figures and images for: Rolling circle amplification (RCA) -based biosensor system for the fluorescent detection of miR-129-2-3p miRNA
Source: PeerJ. 2022 Oct 24;10:e14257. doi: 10.7717/peerj.14257 (PMC9610657; doi:10.7717/peerj.14257)

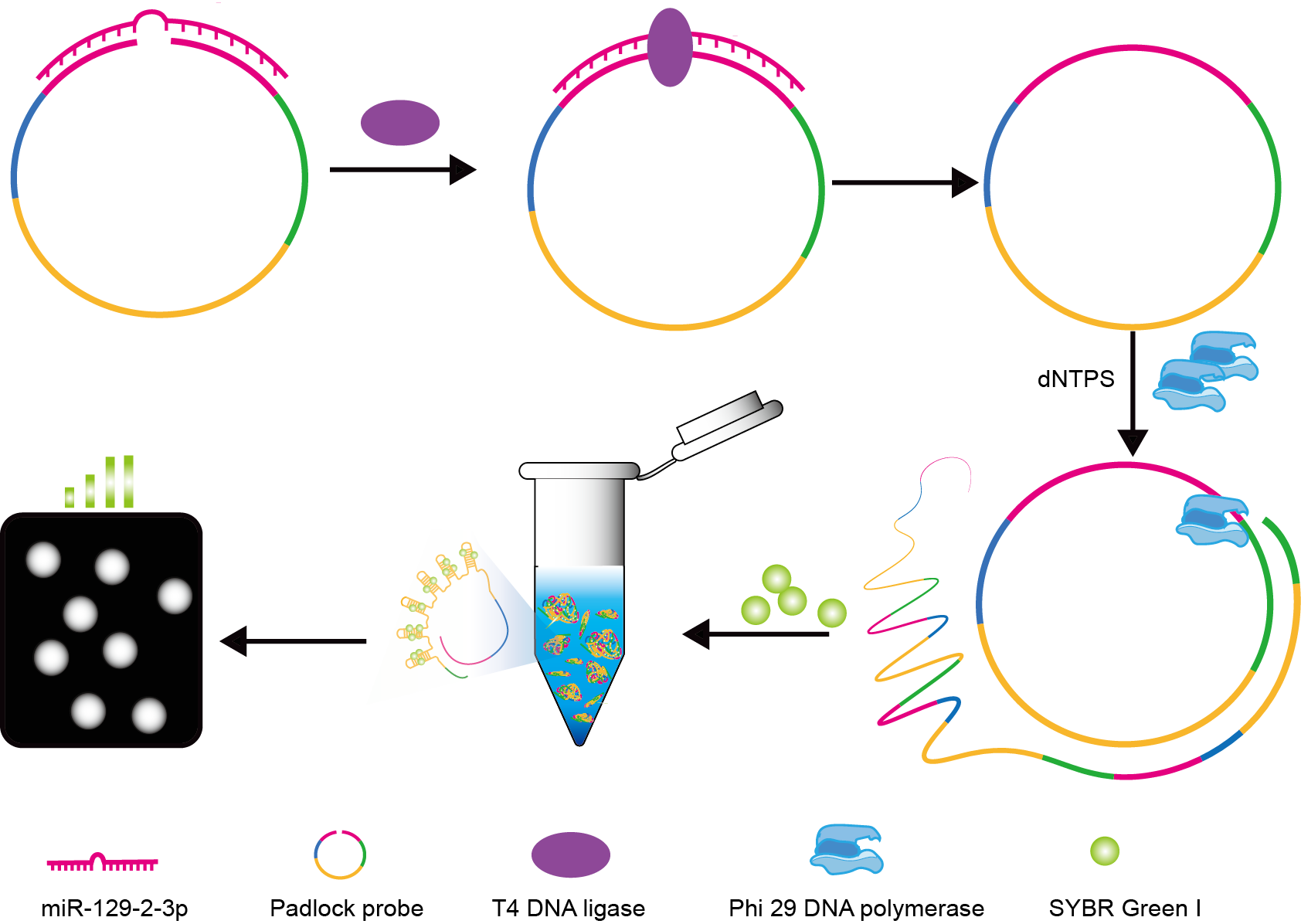

Supplement: Supplemental Information 2 — Hybridization of target to padlock probe by annealing. Circular padlock probe is formed via ligation in the presences of T4 DNA ligase. RCA reaction occurs and target miRNA is amplified to produce a large amount of dsDNA. As a result, dsDNA are able to bind SYBR Green I, then fluorescence signal can be read. [file peerj-10-14257-s002.png]

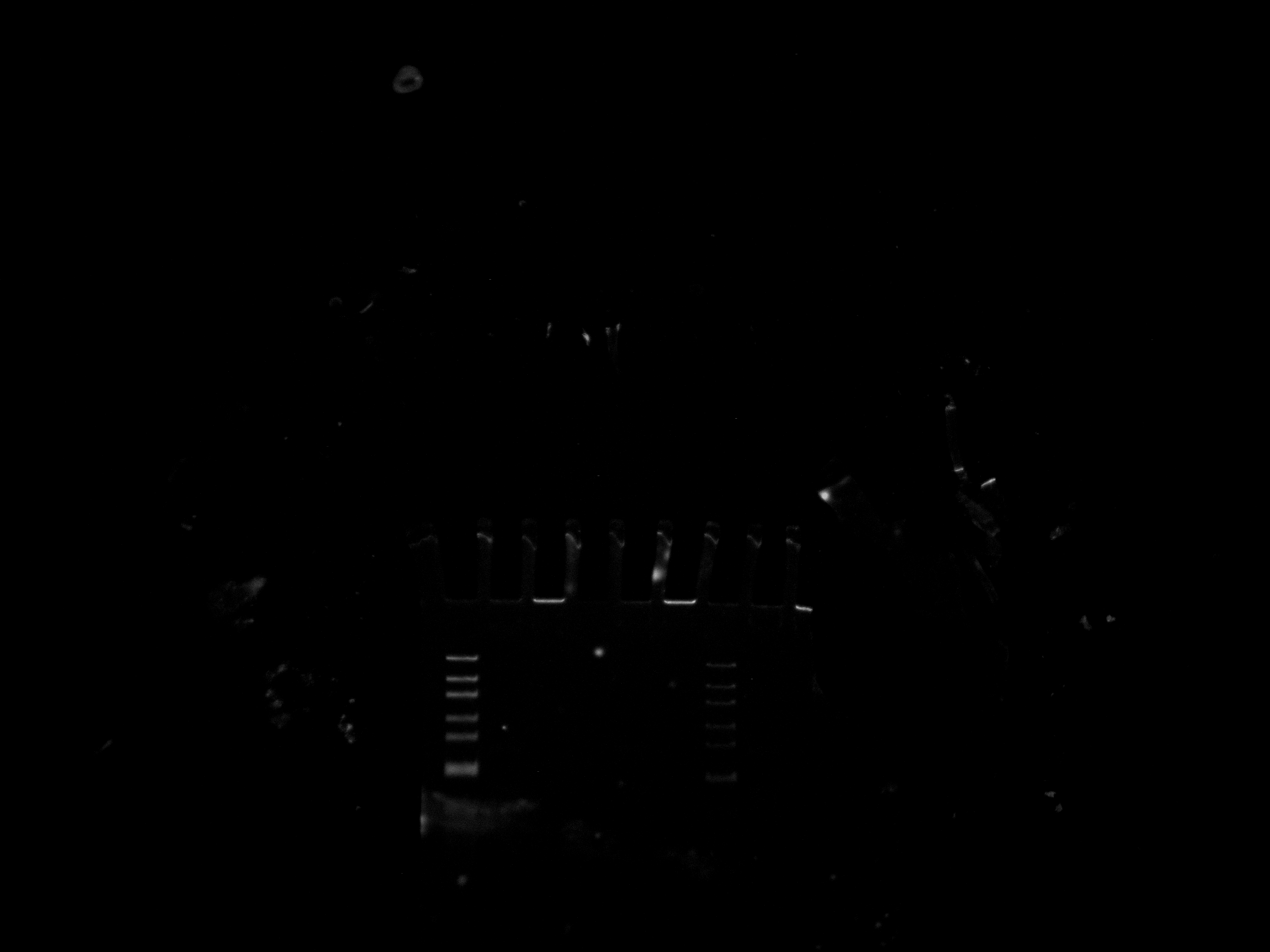

Supplement: Supplemental Information 3 — The feasibility of RCA-based biosensor system. (A) Fluorescence spectra of RCA-based biosensor system in the absence (b) and presence (a) of target miR-129-2-3p; (B) PAGE gel electrophoresis results for the amplification products of absence (a) and presence (b) of target miR-129-2-3p. [file peerj-10-14257-s003.zip › Raw1 for Fig.1/P-RCA▓·╬∩╡τ╙╛═╝.bmp]
